# *Rhus coriaria* extracts inhibit quorum sensing-related virulence and biofilm production in drug-resistant *Pseudomonas aeruginosa* recovered from burn wounds

**DOI:** 10.22038/IJBMS.2022.66085.14527

**Published:** 2022-11

**Authors:** Akhter A Ahmed, Fraidoon A. Salih, Mohammad Yousef

**Affiliations:** 1 Department of Biology, Salahaddin University Erbil, Erbil, Iraq; 2 Ministry of Health, Erbil, Iraq

**Keywords:** Biofilm, Exotoxin A, *Pseudomonas aeruginosa*, Quorum sensing, Sumac

## Abstract

**Objective(s):**

Numerous studies have confirmed sumac’s ability to inhibit pathogens and even eradicate chronic drug-resistant infections. Current research was conducted to demonstrate the action of various sumac extracts at sub-inhibitory concentrations in modulating pathogen-related characteristics instead of killing them.

**Materials and Methods:**

The influence of sumac extracts on the quorum sensing dependent virulence of multidrug-resistant isolates of *Pseudomonas aeruginosa* recovered from burn wounds was considered by detecting the effect on biofilm development, various virulence factors, and expression of bacterial exotoxin A and quorum sensing related genes.

**Results:**

Experiments to characterize and measure sumac extract’s impact on the *P. aeruginosa* growth, biofilm, exoproteases, pyocyanin, motility, and the quorum sensing networks revealed that all studied characteristics were reduced by concentrations below inhibition without affecting bacterial growth. Furthermore, the expression of exotoxin A, *rhl*, and *las* glucons was declined or even inhibited by lower levels of sumac fruit fractions.

**Conclusion:**

The findings revealed that sumac fights infections either by its inhibitory effect on the bacterial cells or by reducing bacterial signaling and virulence by disruption of the bacterial signal system.

## Introduction

Pathogenic bacteria are thought to be the most frequently concerned microorganisms in infectious diseases. Regardless of the innovation of antimicrobial therapy, bacteria develop resistance to these drugs through a variety of mechanisms (1).

The fact that multidrug-resistant microorganisms cause infectious diseases that kill sixteen million people each year necessitates an urgent search for alternative strategies to develop new drugs and drug targets to combat these microbes (2-4). The development of novel appliances to inhibit or reduce the expression of bacterial virulence via blocking bacterial cell-to-cell coordination which is recognized as quorum sensing (QS), is a promising approach to improving unique antipathogenic treatments (5). QS is an intercellular relationship system between microorganisms of the same or different strains that organizes transcriptional regulation of genes responsible for various vigorous functions (1).

Gram-positive and negative bacteria use small molecules of oligopeptides as signals named autoinducers and Lux operons respectively, which are self-inducing molecules that activate in a density-dependent manner to regulate the transcription of certain enzymes involved in QS mediators and consequently bacterial gene expression (6). *Pseudomonas aeruginosa *(*P. aeruginosa*) which is a Gram-negative, adaptable human pathogen that causes several life-threatening infections, uses N-acyl homoserine lactones (AHLs) as QS signal to monitor the population density. The two interrelated QS systems Rhl and Las work in an organized way, they are composed of RhlI and LasI synthetases that produce the auto-inducer signaling molecules N-butanol-l-homoserine lactone (BHL) and N-(3-oxododecanoyl)-l-homoserinelactone (OdDHL), respectively. The other system called the *Pseudomonas* quinolone signal (PQS) has a vital role in the bacterial QS process (7). Many *P. aeruginosa* virulence factors are regulated by the QS mechanisms for example exopolysaccharide, Las A protease, pyocyanin production, Las B elastase, and swarming motility that contribute to the bacterial pathogenesis (8).

Significant efforts have been made to prevent bacteria from becoming resistant to the present antibiotics and, as a result, limit the rise of infectious diseases through anti-QS agents. Among these, the bioactive compounds of medicinal plants are considered interesting in targeting the QS mediators through various mechanisms (9).

Sumac (*Rhus coriaria *L. Anacardiaceae) is a recognized spice that grows in a wide range of places, including the Mediterranean countries, North Africa, South Europe, Iraq, Iran, and Afghanistan (10). It is a well-known, widely used spice that has been extensively used for both medical and other purposes. Because of its high tannin content, its leaves have been applied as a tanning agent. Sumac fruit has diuretic properties and is used to treat bowel complaints and reduce fever. It is also traditionally used as a remedial herb due to its wound-healing properties and antimicrobial activities (11). Several studies reported the inhibitory effect of sumac against Gram-positive bacteria (*Staphylococcus aureus* & *Bacillus spp*.) and Gram-negative pathogens (*P. aeruginosa, Escherichia coli *& *Salmonella enterica*) (11-15). The phytochemical studies of *R. coriaria* stated that the leaves contained phenolic, gallic, benzoic, protocatechuic, and vanillic acid, anthocyanins, and numerous flavonoids as kaempferol glycosides and quercetin (16). The fruit of *R. coriaria* contains flavonoids, phenolic acid, anthocyanins, hydrolyzable tannin, and organic acid. These chemicals have gained interest due to their possible ability to decrease the causes of chronic diseases, strengthening the defenses against free radical species (17). Even though some studies provide encouraging evidence for the efficiency of sumac on bacterial pathogens, there is a further need to explore this issue, particularly its anti-biofilm and anti-QS properties on *P. aeruginosa* isolates. There have been studies on numerous *P. aeruginosa* virulences, but none have addressed the impact of sumac on exotoxin A (ETA) at the level of expression.

The present study was carried out to consider the role of sumac extracts in weakening virulence factors and ETA by affecting the expression of networks of *Rhl* and *Las* genes.

## Materials and Methods

***Plant material***

*R. coriaria* fruit from Akre, was from Kurdistan, Iraq. Chemicals: normal hexane, chloroform, ethyl acetate, n-butanol, ethanol (Scharlau, Sapin), dimethyl sulfoxide (DMSO, Merck Germany) were used as solvents. Azocasein and Pyocyanin production broth (PPB: 2% peptone, 1% K2SO, 0.3% MgCl2) were from Sigma, USA. Bacteriological media: Cetrimide agar (C.A, Acumedia, Neogen, USA), MacConkey (M.A) and Tryptic Soy Broth (T.S.B) Nutrient agar (N.A), Nutrient broth (N.B), Luria-Bertani media (L.B), Mueller Hinton agar (M.H.A), and the antibiotic discs were from Oxoid, UK.

***Extraction process***

 Sumac fruit was collected by gentle rubbing from Akre, Kurdistan, Iraq, in August and identified by the Herbarium of the Biology Department at the College of Science, University of Salahaddin, Erbil, Iraq, and processed using the previously specified methods (18). Briefly, pulverized plant material was fractionated by sonication using various solvents: normal hexane, chloroform, ethyl acetate, n- butanol, ethanol, and water. The solvents were removed from the extracts by vacuum evaporator to obtain each fraction’s crude extract. Each fraction was sterilized by passing through a 0.45 membrane filter after being freshly dissolved in 10% DMSO and stored at -20 ^°^C. To exclude their effect, the solvents were used as both blanks and controls in the whole experimentations throughout the study.

***Specimens collection and samples sources***

Twenty-five non-duplicate *P. aeruginosa* isolates were collected from hospitalized burn patients at West Erbil Emergency Hospital’s burn center. The samples were taken from burn ulcers and exudates with sterile cotton swabs. The specimens were primarily streaked onto M. A. and C.A media and incubated at 37 ^°^C for 24 hr. Many biochemical and conventional diagnostic tests, as previously described (19), were used to identify the distinct colonies as *P. aeruginosa.* VITEK 2 automatic system (Biomerieux, France) was used to confirm the identification of bacterial isolates. The recognized isolates were experienced for their susceptibility to various antibiotics (Amikacin, Chloramphenicol, Ceftazidime, Ciprofloxacin, Doxycycline, Meropenem, Netilmicin, and Tobramycin) and the isolate which was resistant to most tested antibiotics was selected for all assays during the study. The identified bacterial colonies were then subcultured in 1 ml of T.S.B supplemented with glycerol (30%) and stored for further study at -70 ^°^C.

***Minimum inhibitory and bactericidal concentrations (MICs & MBCs)***

A broth microdilution technique was applied to find the minimum inhibitory concentration (MIC) of sumac extracts against multidrug-resistant (MDR) isolates of *P. aeruginosa* (20). In the wells of a 96-well polystyrene microtitre plate(MTP), 10 µl of *P. aeruginosa* cells at stationary-phase (equilibrated to OD550 0.5) were transferred to 100 µl N.B containing a range of extract concentrations (1-30 mg ml-1). At 37 ^°^C, cultures were incubated aerobically for 24 hr. The lowest concentration with no discernible growth was selected as MIC and the latter was confirmed by Elisa reader comparing absorbance before and after incubation. MBC was established by spreading 100 µl samples from wells with no visible growth onto N.A and incubating aerobically for 24 hr at 37 ^°^C. Plates with no visible growth were classified as MBC. Sub-inhibitory concentrations were chosen for further assessment of anti-biofilm and anti-virulence activity. On three separate occasions, three biological samples were analyzed.

***Growth and viability***

*Bacterial growth curve analyses*

The flask incubation method (21) was used to check the anti-QS potential and to show that the test plant fractions have no effect on bacterial growth. An overnight bacterial culture with 1% concentration of MDR *P. aeruginosa* (OD adjusted to 0.5 at 550 nm) was transferred into Erlenmeyer flasks containing 50 ml of L.B broth supplemented with a sub-inhibitory concentration (SIC) of plant extracts. The flasks were incubated for 24 hr at 37 ^°^C with 180 rpm agitation in a rotatory shaker. OD550 was measured at hourly intervals for about 24 hr. Un-inoculated L.B medium was used as a control and the change in optical density was designed over time. The standard error was calculated after analyzing three biological replicas on separate occasions.

***Protease assay***

The assay designated by Chu *et al*. (22) was used to examine the protease activity. Both untreated and treated *P. aeruginosa *were inoculated separately on L.B agar (supplemented with 2% skim milk). After 48 hr of incubation at 37 ^°^C, a clear zone adjacent to the bacterial colonies was recorded as casein proteolysis. On three separate occasions, three biological replications were examined, and the standard error was calculated.

***Azocasein degrading proteolytic activity***

To assess the proteolytic activity, 150 µl of both treated and untreated cell-free supernatant of *P. aeruginosa *culture were added to 1 ml of 0.3% azocasein in 0.05 M TrisHC1 and 0.5 mM CaCl2 (pH 7.5) and incubated for 15 min at 37 ^°^C. The reaction was diminished by trichloroacetic acid (l0%, 0.5 ml) then the tubes were centrifuged, and the absorbance was measured at 400 nm (23). On three separate occasions, three biological samples were analyzed, and the standard error was calculated.

***Pyocyanin assay***

After growth in L.B medium, overnight cultures were adjusted to OD550 of 0.5 and diluted to (1:10) in PPB. A 20 ml sample of the culture containing a SIC of the plant extracts was grown in PPB for 24 hr before being extracted with 3 ml chloroform. The produced blue part was re-extracted in 1 ml of 0.2 M HCl. The absorbance of the resulting red color was measured at 520 nm. The pyocyanin concentration was calculated by multiplying the absorbance value by 17.07 (24). On three separate occasions, three biological samples were analyzed, and the standard errors were calculated.

***Motility assays***

Motility (swimming and swarming) tests were performed as described by Deziel *et al*. (25). Overnight cultures from a single colony were point inoculated onto both swimming (1% tryptone, 0.5% NaCl, and 0.3% agar) and swarming (1% peptone, 0.5% NaCl, 0.5% agar, and 0.5% filter-sterilized D-glucose) plates supplemented with SIC of plant extracts and incubated at 30 ^°^C for 24 hr. Measuring the zone diameter of the motility was considered to record the extent of swarming and swim area. Swim and swarm agar plates with no extracts added were used as controls. On three separate occasions, three biological samples were analyzed, and the standard error was calculated.

***Biofilm assay***

The assay of Limban *et al*. (26) was used to evaluate the impact of plant extracts on bacterial biofilm. Overnight cultures of *P. aeruginosa *were re-suspended in L.B broth in the presence and absence of SICs of the plant extracts and incubated at 37 ^°^C in a static condition for 24 hr. The liquid part in the wells of the MTPs was discarded, and the wells were thoroughly cleaned with phosphate buffer saline (PBS) three times. The wells were dried and stained with crystal violet (1%), then the dye was solubilized by ethanol. The abiotic surface adhesion ability was quantified by reading the OD of the colored suspension with an Elisa reader (Epson, Biotek, UK) at 490 nm. On three separate occasions, three biological replications were examined, and the standard errors were calculated.

***RNA extraction***

To monitor the expression of bacterial ETA both Las and RhI QS genes were targeted. A total RNA extraction kit from Jena Bioscience (Germany) was used to extract the RNA from bacterial cells in the mid-exponential growth phase grown in the control (untreated) and treated with SIC of the extracts. To purify the extracted RNA from remaining genomic DNA, RNase-free DNase I (Promega, USA) was used. The purity and concentration of the extracted total RNA were determined using an IMPLEN Nanodrop spectrophotometer and ultraviolet absorption (260/280 nm).

***Real-time reverse transcription polymerase chain reaction (RT-qPCR)***

The primers used for quantification of *ETA, *5’-GACAACGCCCTCAGCATCACCAGC-3’5’- CGCTGGCCCATTCGCTCCAGCGCT-3’(27) and QS genes were; (sense and antisense):* lasI, *5’-ATGATCGTACAAATTGGTCGGC-3’and 5’-GTCATGAAACCGCCAGTCG-3’ (28); *lasR*, 5’-ATGGCCTTGGTTGACGGTT-3’ and 5’-CAAGATCAGAGAGTAATAAGACCCA-3’ (29); *rhlI, 5’-*TTGGTCATGATCGAATTGCTC-3’ and 5’-ACGGCTGACGACCTCACAC-3’; *rhlR*, 5’-CAATGAGGAATGACGGAGGC-3’ and 5’- GCTTCAGATGAGGCCCAGC-3’(28).

In a 20 µl total reaction volume, a one-step quantitative RT-PCR SuPrimeScript RT-PCR Kit with SYBER Green l (GeNet Bio) and the PCRmax Eco 48 Real-Time PCR system were applied to dignify relative expression. The next reaction process was followed: 50 ^°^C for 20 min (cDNA synthesis), 95 ^°^C for 10 min (initial denaturation), 40 cycles of 95 ^°^C for 15 sec (denaturation), and 60 ^°^C for 60 sec (annealing/extension). Amplifications of real-time PCR were conducted in triplicate. The delta cycle threshold (Ct) method was used to analyze the results, and changes in copy number were taken into account (30). The variance in transcripts was calculated using the comparative Ct method in comparison with the untreated culture (control). 

***High- performance liquid chromatography (HPLC)***

The most effective plant fractions (S-3, S-4, S-5, and S-6) were liquefied in their respective solvents (ethyl acetate/n-butanol/ethanol/water) and clarified using a polyvinylidene fluoride hydrophilic membrane syringe (0.22 m, Hinitmoedia), with 10 L aliquots of the filtrate inserted into the LC-MS/MS system. Shimadzu Nexera model UHPLC combined with Shimadzu LCMS 8040 model triple quadrupole mass spectrometer was used in the qualitative and quantitative analysis of 37 phytochemicals. 

***Statistical analysis***

The assay results were analyzed using GraphPad Prism 8.0 software. One-way analysis of variance (ANOVA) was applied to conclude whether there were significant differences between the untreated (control) and treated bacterial isolates.

## Results

***Inhibition of planktonic P. aeruginosa isolates by R. coriaria extracts and analysis of growth***

The minimum inhibitory concentration of plant extract was detected to determine the SIC to study the effect on bacterial growth and inhibition of QS-regulated functions.

**Figure 1:**
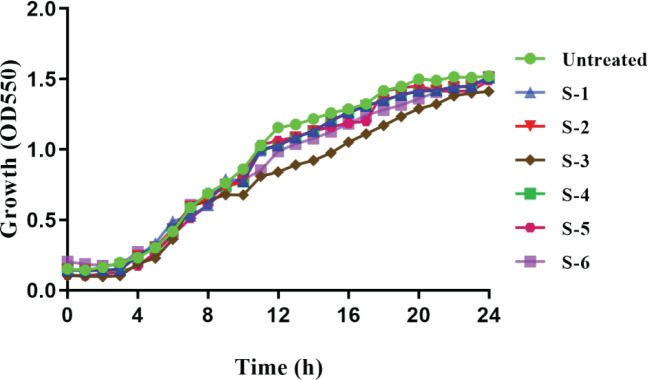
Growth curves of *Pseudomonas aeruginosa* multidrug-resistant (MDR) strain in the presence and absence of *Rhus coriaria* extract over 24 hr. Points represent means from three biological duplications

The MIC and MBC of sumac extracts against the MDR *P. aeruginosa *clinical isolate were determined and the sub-MIC of the extracts (n-Hexane (S-1), chloroform (S-2) (ethyl acetate (S-3), n-butanol (S-4), ethanol (S-5), and water (S-6) used during the study (Table 1). Ethyl acetate fraction (S-3) exhibited the least MIC (2 mg/ ml) followed by butanol (S-4) and ethanol (S-5) fractions which both exhibited MIC of (8 mg/ml). Growth curves with SIC revealed no observable change in the overall cell number and bacterial growth rate (Figure 1) when compared with untreated control points at each time point.

***R. coriaria extracts impact QS-controlled virulence factors***

The influence of sumac extracts on the bacterial virulence factors regulated by QS was considered. The ability of sumac fractions to reduce or inhibit the fabrication of extracellular proteases, which are controlled by the AHL network was studied. When *P. aeruginosa *was cultivated on plates supplemented with skim milk, the addition of extracts virtually reduced the halo area around the bacterial colonies caused by casein breakdown. Untreated plates displayed a 25 mm halo, while colonies on extract plates generated a smaller halo (Table 2), moreover, a significant reduction (*P*<0.0003*)* was indicated in S-3 and S-4. The total activity of protease was also reduced extensively at the SICs of all sumac fractions. The extracts were further tested for their ability to reduce QS-dependent pyocyanin synthesis. Pyocyanin is a blue-green toxic compound exclusive to *P. aeruginosa.* Table 2 shows pyocyanin readings for *P. aeruginosa *cultured in L.B broth with and without SICs of the extracts. Pyocyanin production was reduced significantly (*P*<0.0001) by all sumac extracts, particularly the ethyl acetate fraction (S-3) which recorded the least pyocyanin product. 

***Reduction of P. aeruginosa clinical isolates biofilm formation by R. coriaria extracts without affecting bacterial planktonic growth***

**Figure 2:**
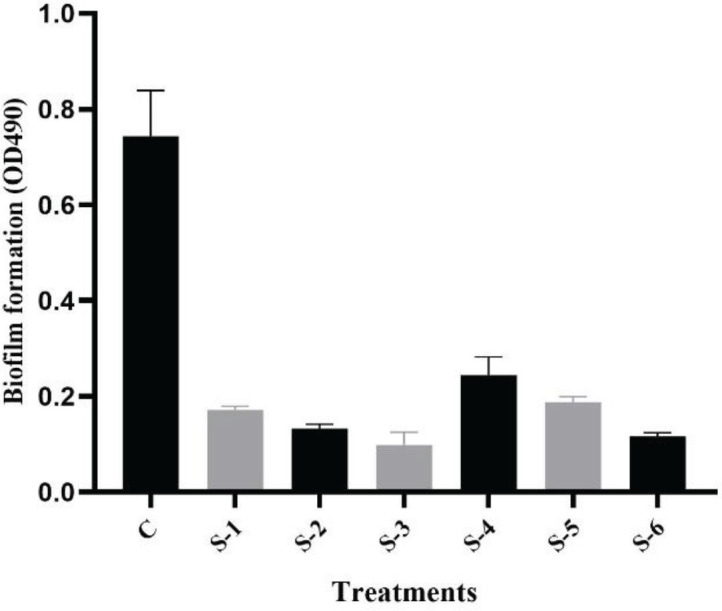
Quantitative measurement of biofilm by *Rhus coriaria* in *Pseudomonas aeruginosa*. The data are shown as the mean±SE of three biological replicas. All data were significant at *P<*0.0001

The capability of sumac extract to decrease or prevent the production of biofilm in the tested *P. aeruginosa *was quantified in 96-well polystyrene microtiter plates. All extracts significantly (*P<0.0001*) reduced the quantity of biofilm in the tested bacteria. Remarkably, the ethyl acetate extract at 1 mg/ml effectively reduced the bacterial biofilm (Figure 2), while the same extract at 2 mg/ml entirely inhibited the biofilm (data not displayed). The findings of biofilm decline are positively consistent with a decline in both investigated motilities (Table 2) as these motilities play a vital role in biofilm attachment and maturation. Unlike antimicrobial agents, potential antibiofilm or anti-virulence agents should not affect growth, as this may result in resistance of bacteria.

***Repression of QS genes by sumac extracts***

**Figure 3:**
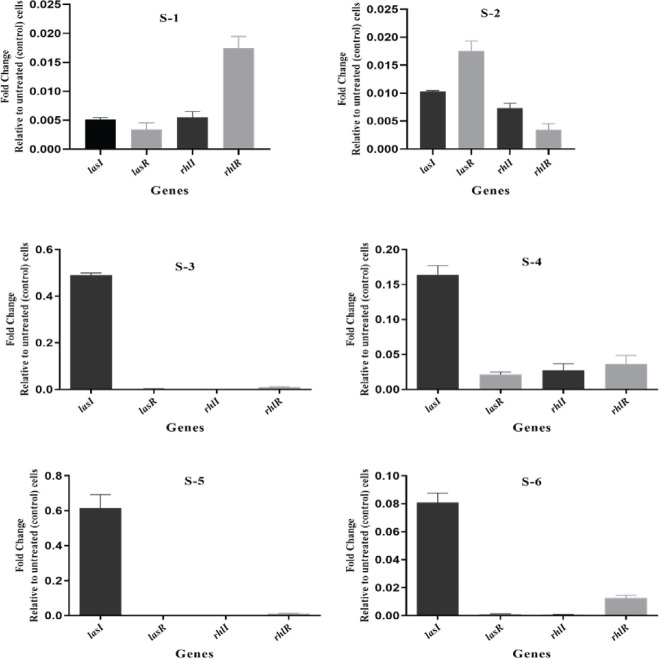
Transcriptional profiles of QS gene expression of treated *Pseudomonas aeruginosa* with sub-inhibitory concentrations of *Rhus coriaria* fruit extracts. RT-qPCR was used to measure the transcription

**Figure 4:**
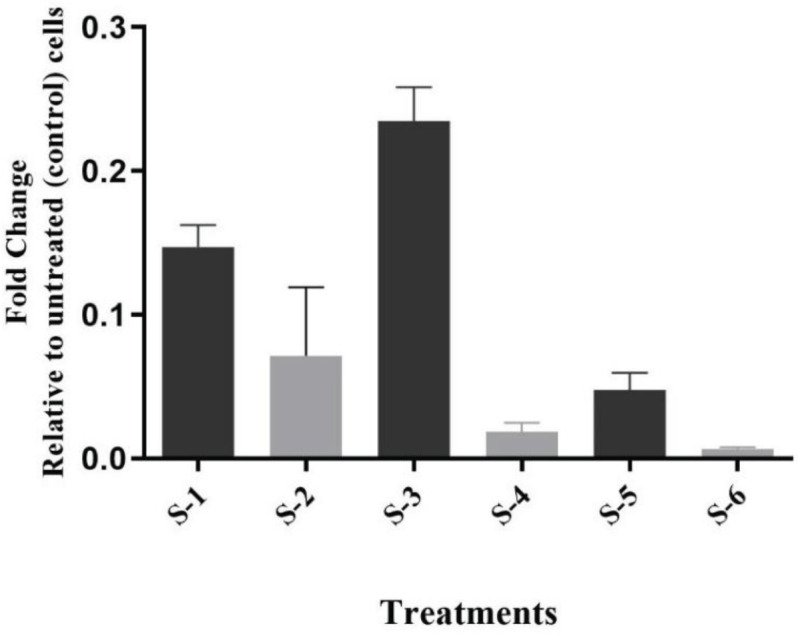
Transcriptional profile of *ETA* gene expression of treated *Pseudomonas aeruginosa* with sub-inhibitory concentrations of *Rhus coriaria* fruit extracts. RT-qPCR was used to measure the transcription. S-1: n-hexane, S-2: chloroform, S-3: ethyl acetate, S-4: n-butanol, S-5: ethanol, S-6: aqueous extract

To show whether the efficacy of sumac fractions on the virulence of *P. aeruginosa *was the consequence of QS inhibition, the expression of the reporter genes (*rhlI*, *rhlR, lasI*, and *lasR*), which are convoluted in the bacterial virulence and biofilm development and *eta* gene was monitored by RT real time-PCR. QS networks are thought to be the most appealing non-lethal marks for antimicrobial remedies. Because eliminating them would reduce bacterial virulence while potentially avoiding the stress that conservative antibiotics exert. Ct values were standardized between biological samples, and changes in copy number relative to untreated control bacterial cells were explored. The expression of the studied genes was expressively reduced in bacterial cells treated with extracts, while growth was unaffected (Figures 3 & 4). Both ethyl acetate (S-3) and ethanol (S-5) sumac fractions effectively reduced the expression of the studied genes. 

All extracts similarly down-regulated the expression of ETA. The aqueous fraction of sumac fruit (Figure 4) was the most effective. These findings supported the current study’s findings of total virulence inhibition. The observed suppression of genes and signal molecules in the las and rhl operons, as well as effects on downstream virulence factors, show that QS networks have a broad influence.

**Figure 5:**
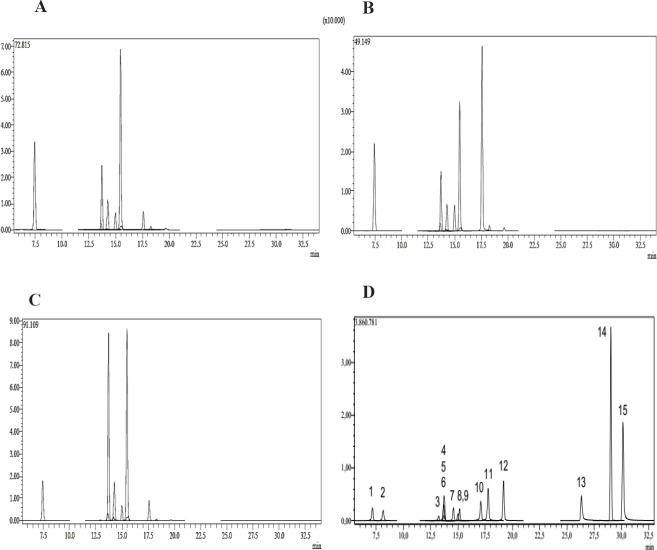
Analytical parameters, description, and quantification of phytochemicals extracted from *Rhus coriaria* using LC-MS/MS

Liquid chromatography Mass spectrometry (LC-MS/MS) analysis was conducted for the most effective fractions of *R. coriaria* fruit using LC combined with MS/MS, which is an ideal analysis of complex mixtures of constituents. The HPLC results showed several compounds’ occurrence in the fractions investigated. According to the results, quercitrin, protocatechuic acid, hyperoside, quercitin, and luteolin-7-glucoside were found differently in the fractions of *R. coriaria. *The maximum content of quercitrin was quantified in all fractions (Table 3 & Figure 5). 

**Table 1:** Minimum inhibitory, sub-inhibitory and minimum bactericidal concentrations of *Rhus coriaria* extracts against multidrug-resistant (MDR) isolates of *Pseudomonas aeruginosa*

Extracts	MIC (mg/ml)	SUB-MIC (mg/ml)	MBC (mg/ml)
S-1	125	100	150
S-2	50	25	75
S-3	2	1	4
S-4	8	4	16
S-5	8	4	16
S-6	16	8	16

S-1. n-Hexane; S-2 Chloroform; S-3, Ethyl acetate, S-4 n-Butanol; S-5 Ethanol; S-6 Aqueous extract

**Table 2:** Effect of Sub-MICs of extracts of *Rhus coriaria* fruit on *Pseudomonas* *aeruginosa* virulence factors

Pseudomonas aeruginosa	Total protease activity	Exoprotease	Pyocyanin production	Motility Swarming Swimming	
Untreated	0.706 ± 0.014	25.33±0.88	5.317 ± 0.044	36.7 ±3.28	54.33±1.20
S-1	0.3±0.009	11.2±0.601	2.4±0.144	11±0.29	32±1.2
S-2	0.18±0.003	6.5±0.289	2.8±0.007	13±0.33	26±0.67
S-3	0.11±0.005	1.2±0.60	1.1±0.024	11±0.17	16±0.88
S-4	0.23±0.012	1.7±0.88	1.5±0.021	11±0.88	22±1.5
S-5	0.17±0.008	19±1.2	1.6±0.041	8.5±0.29	20±0.76
S-6	0.22±0.01	12±0.88	0.62±0.026	5.5±0.5	13±0.17

Data represent mean values of three distinct experiments. All data were significant at *P<*0.0001except exoproteases in S-5 (*P<*0.0003). The activity of total protease is denoted as the absorbance at OD 420; exoprotease is represented in millimeter of the diameter, pyocyanin levels were expressed as microliter of pyocyanin produced per milliliter, swimming and swarming motilities were stated as the diameter in millimeter

S-1: normal-hexane, S-2: chloroform, S-3: ethyl acetate, S-4: n-butanol, S-5: ethanol, S-6: aqueous extracts

C: untreated, S-1: n-hexane, S-2:chloroform, S-3: ethyl acetate, S-4: n-butanol, S-5: ethanol, S-6: aqueous extract

S-1: n-hexane, S-2: chloroform, S-3: ethyl acetate, S-4: n-butanol, S-5: ethanol, S-6: aqueous extract

**Table 3:** Analytical parameters of the LC-MS/MS method for the analyses of 15 phytochemicals extracted from *Rhus coriaria*

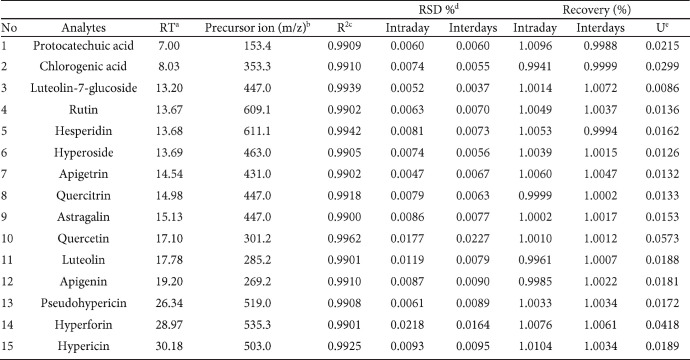

^a ^RT Retention time

^b ^Precursor ion(m/z) Molecular ions of standard compounds

^c ^R2 Correlation coefficient

^d ^RSD Relative standard deviation

^e ^U (%) Percent relative uncertainty at 95% confidence level (k=2)

a:ethyl acetate, b:n-butanol, c:ethanol, d:aqueous extract

The identified 15 phytochemical standards: 1)Protocatechuic acid, 2)Chlorogenic acid, 3)Luteolin-7-glucoside, 4)Rutin, 5)Hesperidin, 6)Hyperoside, 7)Apigetrin, 8)Quercitrin, 9)Astragalin, 10)Quercetin, 11)Luteolin, 12)Apigenin, 13)Pseudohypericin, 14)Hyperforin, 15)Hypericin

## Discussion

A tremendous increase in the incidence of MDR pathogens has motivated studies to search for new alternative strategies. A new approach is weakening bacterial virulence via modulating QS targets instead of bacteriostatic and bactericidal strategies (31). More specifically, the effect on the genes that regulate bacterial virulence activation is of interest to combat microorganisms resistant to conventional therapies. Recent years have seen extensive use of this novel non-antibiotic therapy, which can suppress the expression of bacterial genes, prevent infection, and lower the chance of drug resistance in bacterial cells (32). 

*P. aeruginosa* is recognized as the most critical bacterial pathogen causing considerable morbidity and mortality and has become a serious issue in nosocomial infections, especially in patients with impaired immune systems (33). The universal prevalence of MDR *P. aeruginosa* is increasing. This serious pathogen is naturally resistant to many antibacterial agents, including the majority of β-lactams (34). Moreover, it also has a notable ability to develop or acquire new antibiotic resistance mechanisms. The World Health Organization has considered it among the global priority bacterial pathogens (35), therefore, a unique strategy is required to fight this MDR pathogen. 

Novel strategies are becoming progressively obvious to natural products’ impact on the virulence of the pathogen. The current research characterizes the antibacterial and anti-QS activity of sumac fruit using SICs to test systematically the influence on living bacterial cells. Our tactic extensively diverges from the use of concentrations that kill bacteria and exhibits a diverse line of research concerning the natural product’s extraordinary capability to fight microbes at lower concentrations.

In this study, different extracts of the sumac fruit were chosen as inhibitors and fractionated by solvents (normal-hexane, chloroform, ethyl acetate, n-butanol, ethyl alcohol, and water). The current study’s findings emphasize that all screened fractions are capable to decrease or impede the expression of various virulence factors. It is reassuring to know that our local sumac affects MDR isolates at lower concentrations than documented for bactericidal effects. To ascertain the anti-QS effect, the ability of studied fractions to affect the QS-related virulence was assessed. *P. aeruginosa *uses AHLs as QS signaling (rhl and las complexes) which control bacterial virulence like exotoxins, pyocyanin, exoproteases, and formation of biofilms (36). *P. aeruginosa* secretes exoproteases that significantly contribute to pathogenesis, devastate host tissues, and enhance the bacterium’s proliferation and invasiveness. Their secretion, production, and typical cooperative behaviors are under the control of the QS organization (37). Exoproteases are regulated by las operon, which is recognized to be controlled by lasR and Mvfr systems (25). Significant inhibition (*P*<0.0001) of proteases was perceived in the treated isolates, particularly the butanol and ethyl acetate fractions. *P. aeruginosa* produces a secondary metabolite, pyocyanin, a blue-green toxic substance that degrades the neutrophil-mediated host defense (38). The decreased synthesis of pyocyanin is congruent with the notable decrease in the expression of the* rhl *and* las* networks. Our data on the reduction of proteases and pyocyanin are in line with the findings of Ahmed and Salih (3), who concluded the anti-virulence and anti-QS system of honey. Swarming motility which is driven by bacterial flagella is a QS-dependent virulence function that is crucial for the cell to surface attachment and development of biofilms (39). The decrease in the swarming and swimming movement demonstrates the studied substances’ ability to inhibit flagellar synthesis. Similar consequences were recorded by Mohabi *et al. *(40) who reported that plant extracts reduce both motilities in *P. aeruginosa *significantly besides the reduction effect on QS genes. Biofilm is a community of microbes that lives on surfaces and is reinforced by the formation of a protective and adhesive matrix; biofilms are common in the natural world, the medical field, and other settings. The innate antimicrobial tolerance and resistance have significant effects on infections associated with healthcare (41). Biofilm cells are more resistant to antibiotics and require higher doses of antibiotics to remove when compared with planktonic cells. (4). The results of the present study emphasize that all sumac’s fractions at their SICs could reduce biofilm improvement and the expression of virulence and QS genes without affecting growth. These findings are in line with previous reports (12, 42, 43).

Because QS inhibition is widely recognized as an encouraging strategy for controlling infections due to *P. aeruginosa*, the ability of natural constituents and non-native counterparts to block AHL and their receptors’ binding has been more frequently targeted (44). The quantitative study of the gene expression demonstrated that the SICs of the sumac extracts could affect the studied genes differently through reduced expression**. **Our results confirmed that both QS (*rhl *and *las*) paths that structure the AHL network are down-regulated. This endorses that our extracts influenced the virulence of *P. aeruginosa *through the suppression of QS systems function. *P. aeruginosa *produces some toxins such as ETA which adhere to and damage the host tissues. It is a bacterial enzyme that belongs to the mono-ADP-ribosyl-transferase family and is secreted by most *P. aeruginosa* clinical strains (45). The current study assessed the effect of sumac extracts at their SICs on the expression of ETA, and the obtained data showed that all fractions affect the *eta* gene at the level of expression. Likewise, all investigated fractions declined or repressed the expression of ETA differently; both butanol and aqueous fractions were among the most effective substances used in the current study. This result is somewhat impressive because it is critical for the toxin to be present in sufficient concentration in the environment for effective killing. We have previously reported similar results using natural products like *Quercus* gall extracts and low concentrations of honey (3, 43).

Even though the modes of action of the medicinal plant extracts are multifaceted, a general reduction of the QS function with each of the fractions studied was perceived. This impact can be explained in two ways: the first is due to diverse biochemical compounds in the plants, which may affect the QS functions in different facets. The second clarification is that the effect could not be directly on the main QS systems but the total QS regulators are targeted, such as *Vfr *(46) or *GacA *(47). However, if both main (rhl and las) signaling systems are disrupted, the bacterium may be unable to rebuild the bacterial QS-coordinated virulence factors. This might effectively reduce the virulence and then high mortality allied with *P*. *aeruginosa. *Their impact on the different QS-controlled virulence factors means that sumac extracts affect the expression of the related genes. This impact would designate important interaction of sumac extracts with the *P. aeruginosa* QS systems. 

The ability of most investigated fractions to fight infections may be endorsed by the secondary metabolites they produce. Plant extracts are typically composed of a variety of bioactive compounds or phytochemicals with varying polarities. Because of these factors, their separation remains a significant challenge for the identification and characterization processes. The most active extracts were further analyzed to identify the phytochemical constituents by HPLC. HPLC is a versatile, robust, and novel method for isolating natural products and screening herbal compositions for pharmaceutically active components in medicinal plants (48).

The results of HPLC showed that fifteen different phytochemicals of flavonoid and phenolic compounds are present in *R. coriaria* fruit. Quercitrin, protocatechuic acid, hyperoside, quercitin, and luteolin-7-glucoside were found differently in the studied fractions*. *The maximum quercitrin content was determined in all fractions. Based on the distinct HPLC patterns of the solvent fractions, it is suggested that the sumac fruit contains numerous active constituents and they likely function through different mechanisms. Hence, we lack sufficient data to conclude the mode of quorum quenching, more research is requisite to understand the effects of the investigated plant’s pure active fraction. Consequently, finding novel therapeutic molecules and developing compounds with QS inhibition traits might be conceivable**.**

## Conclusion

The present study validated the direct inhibitory activities of *R. coriaria* fruit fractions against *P. aeruginosa*, which were established by morphological and structural studies equally. Some genes were down-regulated in response to exposure to different fractions of sumac fruit. Bacterial QS molecules and ETA production were reduced remarkably by SICs of the extracts. 

The prospective ability of different sumac extracts to reduce the expression of these studied genes is impressive for the therapeutic and prophylactic application of remedial plants. These consequences afford a novel report that different extracts from *R. coriaria *affect MDR *P. aeruginosa* with paired mechanisms, which implicate the direct inhibition of bacterial growth and modulation of several virulence-coordinated genes.

## Authors’ Contributions

AA and FA Designed the research; AA and MY Conducted the research, AA Analyzed the data and wrote the paper; FA Edited the paper; AA, FA, and MY had primary responsibility for the final content. All authors read and approved the final manuscript.

## Conflicts of Interest

The authors declared no conflicts of interest in the present manuscript.
